# Video laparoscopic intervention for an interstitial pregnancy after failure of clinical treatment

**DOI:** 10.1590/S1516-31802012000300011

**Published:** 2012-07-12

**Authors:** Nilson Abrão Szylit, Sérgio Podgaec, Evelyn Traina, Rita de Cassia Sanches Oliveira

**Affiliations:** I MD, MSc. Volunteer at Birth Control Outpatient Clinic of Instituto Israelita de Responsabilidade Social Albert Einstein, São Paulo, Brazil.; II MD, PhD. Gynecological Clinic, Hospital das Clínicas da Faculdade de Medicina da Universidade de São Paulo, São Paulo, Brazil.; III MD, PhD. Obstetrician and Gynecologist, Mother and Child Department, Hospital Israelita Albert Einstein (HIAE), São Paulo, Brazil.; IV MD PhD. Head of Fetal Medicine, Mother and Child Department, Hospital Israelita Albert Einstein (HIAE), São Paulo, Brazil.

**Keywords:** Pregnancy, ectopic, Laparoscopy, Ultrasonography, Prenatal care, Methotrexate, Gravidez ectópica, Laparoscopia, Ultrassonografia, Cuidado pré-natal, Metotrexato

## Abstract

**CONTEXT:**

Interstitial pregnancy is a rare form of ectopic pregnancy for which the best therapeutic course of action has yet to be determined. Surgical intervention entails a high risk of hemorrhage due to the great vascularization of the cornual region of the uterus. Case descriptions facilitate the analysis of results and aid clinicians in determining the most appropriate course of action in these situations.

**CASE REPORT:**

In a patient with an ultrasound diagnosis of interstitial pregnancy, clinical treatment using methotrexate was chosen. However, after one week, there was a marked decline in the serum level of the b subunit of chorionic gonadotropin hormone, although an ultrasound examination revealed embryonic cardiac activity. A second dose of the chemotherapy was administered. Embryonic cardiac activity persisted 48 hours later. Video laparoscopy was performed to achieve right-side cornual resection, which resulted in satisfactory resolution of the case.

## RESUMO

**CONTEXTO:**

A gestação intersticial é uma forma rara de gestação ectópica. A melhor opção terapêutica ainda não está definida, havendo risco de hemorragia com o tratamento cirúrgico, em decorrência da grande vascularização na região cornual uterina. A descrição de casos facilitam a análise dos resultados e ajuda os clínicos a determinarem a melhor conduta nessas situações.

**RELATO DE CASO:**

Em paciente com diagnóstico ultrassonográfico de gestação intersticial, optou-se por tratamento clínico com administração de metotrexate. Após uma semana, houve declínio acentuado do nível sérico da sub-unidade b do hormônio gonadotrófico coriônico, porém, na ultrassonografia, foi detectada a presença de atividade cardíaca embrionária. Foi administrada uma segunda dose do quimioterápico. Após 48 horas, a atividade cardíaca persistia. Realizou-se vídeo-laparoscopia para ressecção cornual direita, com resolução satisfatória do caso.

## INTRODUCTION

Interstitial pregnancy is a rare form of ectopic pregnancy that occurs when a blastocyst implants between the ostium and the isthmic region of the fallopian tube, where the tube transverses the muscle wall of the uterus.^1^ Interstitial pregnancy should not be confused with cornual pregnancy, which develops in the horn of a bicornuate uterus.^2^ Interstitial pregnancy accounts for 2 to 4% of all ectopic pregnancies and has a mortality rate of 2.0 to 2.5%. Accordingly, interstitial pregnancy is the main cause of maternal death in the first trimester of pregnancy, and its associated risks are approximately seven times greater than the risks of ectopic pregnancies in general.^3^^,^^4^^,^^5^^,^^6^ This high maternal mortality rate can be attributed to the possibility of rupture. If this occurs, it results in catastrophic hemorrhaging because of the extensive vascularization of the region.^2^ The treatment for interstitial pregnancy remains controversial. Conservative treatment with methotrexate is an option for patients with a confirmed diagnosis who wish to preserve their reproductive function, provided that there has not been any occurrence of uterine rupture.^7^ Late diagnosis diminishes the viability of conservative treatment or minimally invasive surgery, and increases the maternal mortality risk.^6^^,^^8^

## CASE REPORT

The patient was 33 years old and had a history of having delivered a baby by caesarian section 23 months before the procedure reported here. Six months prior to that delivery, she had undergone laparoscopic myomectomy of the right cornual region, excision of ovarian cysts on the left side and resection of the terminal ileum, cecum and appendix because of endometriosis. The patient had not undergone any reproductive treatments. Given the characteristics of the case and the requirements under the code of conduct, the protocol did not require submission of this study to the ethics committee at Hospital Israelita Albert Einstein (HIAE).

**Figure 1.**
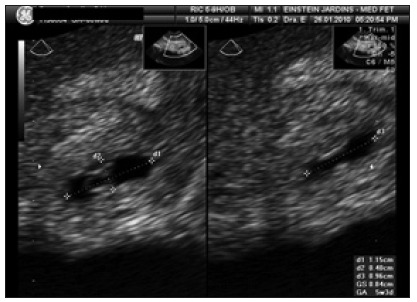
Gestational sac at time of diagnosis of interstitial pregnancy.

The patient presented a five-day menstrual delay, b-fraction human chorionic gonadotropin (b-hCG) concentration of 11,631 mIU/ml, and did not show any clinical signs such as pelvic floor pain or vaginal bleeding. Transvaginal ultrasonography revealed an empty uterine cavity and the presence of an oval entity between the endometrial cavity and the extrauterine tubular region. A thin rim of myometrial tissue (cross-sectional area, 17 mm x 15 mm) encircling an echogenic center (11 mm x 4 mm x 9 mm) suggestive of a gestational sac with an interior vitelline vesicle 2.3 mm in diameter was observed (Figure 1), and Doppler velocimetry indicated peripheral flow. These findings corresponded to a gestation period of 5.5 weeks.

**Figure 2.**
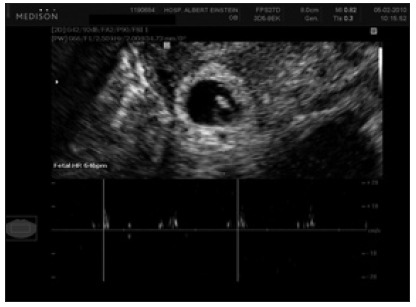
Embryonic cardiac activity in a case of interstitial pregnancy.

Serial measurements of serum b-hCG concentrations showed an increase to 21,281 mIU/ml, 48 hours later. In such cases, an intramuscular injection of methotrexate at a dosage of 50 mg/m^2^ of body surface area is recommended in the treatment protocols used for extrauterine pregnancies.^7^ In this case, a 96-mg dose, corresponding to the patient’s height of 1.76 m, was used. One week later, the patient’s serum b-hCG concentrations showed a significant decrease (to 18,144 mIU/ml), and the patient remained asymptomatic. However, routine transvaginal ultrasonography revealed a small increase in the size of the gestational sac and embryonic cardiac activity (Figure 2).

**Figure 3.**
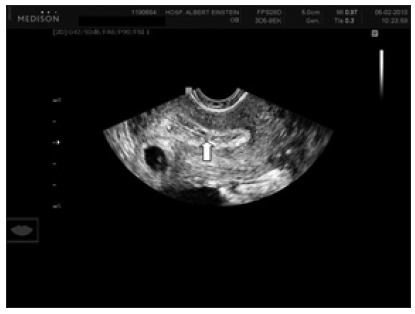
Gestational sac next to endometrium in a case of interstitial pregnancy. White arrow shows the empty uterine cavity.

**Figure 4.**
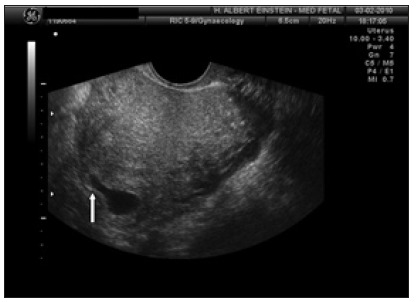
Gestational sac growing into the fallopian tube (white arrow).

**Figure 5.**
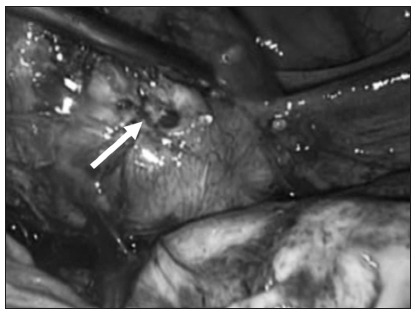
Laparoscopic image. White arrow shows gestational sac.

Given the abovementioned findings, a decision was made to give a second intramuscular injection of methotrexate, with ultrasonographic control 48 hours later. The patient’s serum b-hCG concentrations continued to decrease (to 14,212 mIU/ml), but embryonic vitality persisted. The gestational sac increased longitudinally in size, in the direction of the isthmic portion of the fallopian tube (Figure 3 and 4). After discussing the risks associated with progression of the gestational sac, such as rupture of the fallopian tube, the couple in question opted for video laparoscopy to resect the right-side section of the cornua (Figure 5). The procedure was completed without any complications or significant bleeding. The patient’s preoperative hemoglobin (Hb) level was 11.5 g/dl and her hematocrit (Ht) level was 35.6. Her Hb and Ht levels were 11.1 g/dl and 34.7, one day after this procedure. No complications occurred, and the patient made a very good recovery. She was released two days after the procedure.

## DISCUSSION

The isthmic portion of the fallopian tube, which transverses the uterine wall, is the segment that commences at the ostium of the fallopian tube and ascends laterally to the exterior of the uterus. It measures 0.7 mm in diameter and 2 cm in length, and is the region where the tube is enclosed by a thin film of uterine muscle tissue. The anatomy of the isthmic portion of the fallopian tube is thought to enable greater expansion than is possible in other tubular regions. As a consequence, interstitial pregnancy can persist, in rare cases, asymptomatically for up to 16 weeks.^1^

On the other hand, recent evidence has demonstrated that rupture in an interstitial pregnancy always occurs before 12 weeks.^1^ Other forms of ectopic pregnancy present similar rupture time ranges, namely, seven to nine weeks.^9^ In two reports relating to interstitial pregnancy, it was concluded that the average gestation periods at the time of the diagnosis were 6.9 ± 0.3 weeks^1^ and 8 ± 2 weeks,^10^ respectively. These findings were similar to what was reported from a prospective study on 119 cases of ectopic pregnancy that were not interstitial (6.8 to 8.8 weeks gestation).^11^

The patient whose case is reported here had undergone uterine surgery and appendectomy because of endometriosis: both of these were risk factors that supported investigating the possibility of an ectopic pregnancy. High-resolution transvaginal ultrasonography is the primary method used to diagnose ectopic pregnancy, thus enabling diagnosis prior to uterine rupture. Three-dimensional transvaginal ultrasonography has some advantages over two-dimensional transvaginal ultrasonography, in that it enables viewing of the cornual area of the uterus, which facilitates differentiation between an interstitial and a corneal gestational site.^12^^,^^13^^,^^14^^,^^15^

Early diagnosis makes conservative treatment with methotrexate feasible, which avoids the risks associated with surgical procedures and preserves reproductive functions. Local or systemic administration of methotrexate, or a combination of the two, can be used. The course of treatment most often used is one or two cycles of intramuscular injection of methotrexate. The success rates of local injection and of combined or systemic treatment vary between 62.5 and 83%.^1^^,^^2^^,^^16^^,^^17^ Although many authors have recommended a particular serum β-hCG concentration as a criterion for proceeding with conservative treatment using methotrexate, there is no consensus in the literature regarding the safety and expected outcome of this method.^1^ Generally, cases of interstitial pregnancy are associated with elevated serum β-hCG levels.^7^

Tulandi and Al-Jaroudi^1^ reported that in three patients for whom methotrexate treatment failed, the serum β-hCG levels ranged from 600 to 13,420 mIU/ml. Given this wide range, serum β-hCG levels may not be a good predictive measurement of success in clinical treatment for cases of interstitial pregnancy. In another study, the success rate of systemic methotrexate treatment for interstitial pregnancy exceeded 80% in patients with serum β-hCG levels up to 106,634 IU/l, even when an embryonic heartbeat was present.^18^

The medical course of action in cases of interstitial pregnancy should always be personalized for each patient. Systemic treatment with methotrexate via single or multiple intramuscular injections is used in cases in which the embryo does not show cardiac activity. It is interesting to note that methotrexate treatment diminishes the proliferation of the trophoblast, even though it fails to cause embryonic death.^2^ When cardiac activity is observed, the recommended treatment is an intra-amniotic injection of potassium chloride and methotrexate, guided by transvaginal ultrasonography.^1^^,^^7^^,^^13^ In the case reported here, the initial treatment chosen was a single intramuscular injection of methotrexate. This treatment has the advantages of generally being highly effective, not causing any side effects and avoiding possible complications associated with a surgical procedure.^18^ One week after the initial dose, the patient’s serum β-hCG concentration had decreased by over 15%, which is considered to be sufficient decline on the seventh day after treatment.^7^ Nevertheless, transvaginal ultrasonography examination detected an embryo with cardiac activity.

A second dose of methotrexate is recommended when serum β-hCG levels do not decrease properly. In a review on 20 cases, a second dose was required in 30% of them, with a success rate of 94%.^18^ After conservative treatment with methotrexate, it is necessary to continue making regular examinations on the patient, because of the risk of rupture, and to measure serum β-hCG levels to assess whether the treatment was successful. Serially repeated transvaginal ultrasonographic examinations are not necessary, since the differences that can be detected in such examinations are not indicative of, or able to predict, treatment failure.^18^^,^^19^

Nevertheless, the evidence of embryonic cardiac activity one week after the initial treatment, despite a significant decrease in serum β-hCG levels, showed that one follow-up transvaginal ultrasonographic examination was indispensable in our case. In the present case, the option of repeating the systemic dose was chosen, with a follow-up transvaginal ultrasonography, in the hope that embryonic cardiac activity would cease spontaneously. This approach has been shown to be successful in other cases described previously in the literature.^1^^,^^13^

When embryonic cardiac activity is present, local injection of 2 mEq/ml potassium chloride with 1 mg/kg of methotrexate into the gestational sac, guided by transvaginal ultrasonography, produces good results.^1^^,^^2^^,^^7^^,^^20^^,^^21^ Although a local injection is relatively safe and effective, it does require extensive technical training and experience. The cornual region is highly vascularized and there is a risk of a puncture-related hemorrhage.^9^ Additionally, some authors have reported side effects in more than 40% of patients treated with multiple doses.^22^

Regarding surgical treatment, the most notable immediate risk is hemorrhage during surgical manipulation of the myometrial tissue.^1^^,^^13^ Considering that, in the present case, embryonic cardiac activity persisted even after the second treatment with methotrexate, the risks and benefits of a local injection of potassium chloride and methotrexate and the option of laparoscopy were discussed with the couple. The couple opted for minimally invasive video laparoscopic surgery. This procedure preserves the patient’s fertility and also enables fast recovery that allows the patient to resume regular activities quickly. In the present case, it is possible that the successful laparoscopic resection with minimal blood loss that was achieved can be attributed to the preceding treatment with methotrexate, which had attenuated trophoblast development.^23^ Treatment of interstitial pregnancy using methotrexate in conjunction with embolization of the uterine arteries, with transcervical suction under laparoscopic and hysteroscopic guidance and with dactinomycin as second-line chemotherapy has also been reported recently.^24^^,^^25^^,^^26^^,^^27^^,^^28^

The most appropriate treatment for interstitial pregnancy remains undefined and always requires individualization for each patient. The choice of treatment will depend on the initial serum β-hCG levels and whether an embryo is present, as well as the local resources available and the experience of the medical team. Depending on each patient’s clinical profile, and provided that there is no imminent risk of rupture, treatment with methotrexate can be considered as a first line of treatment. It should, however, be followed by ultrasonographic examination. The decline in serum β-hCG levels cannot be used as the sole measurement of success, since it does not guarantee involution of the pregnancy. Video laparoscopy remains a safe and efficient treatment option for interstitial pregnancy when pharmacological treatment is unsuccessful.

Since occurrences of interstitial pregnancy are rare and the options for treatment decisions remain open, we carried out a review of the literature in order to complete this report. In PubMed, we searched for the term “pregnancy, ectopic” in the medical subject headings, and the term “interstitial” in all words. The date range for this search was from July 31, 2005, to July 30, 2010, in the English or Portuguese languages. In Lilacs (Literatura Latino-Americana e do Caribe em Ciências da Saúde), we searched for the term “gravidez ectópica” in the subject and “intersticial” in all words. The date range for this search covered five years prior to July 30, 2010. In the Cochrane Library, we searched for the expression “interstitial pregnancy”. The date range for this search covered five years prior to July 30, 2010. In the Embase database, we searched for the expression “interstitial pregnancy” in “all fields”. The date range for this search covered five years prior to February 23, 2011.

In PubMed, we found 55 papers, and 20 of them related directly to treatment. In Lilacs, we found only one paper (in Portuguese), which had already been selected from the PubMed database.^7^ In the Cochrane Library, we found seven references, which all dealt with other types of ectopic pregnancy. In the Embase databases, we found 45 references. Four of these were directly pertinent to our study: two case reports,^23^^,^^24^ one prospective study^22^ and one review.^7^ This last review had already been selected from the PubMed database. A descriptive summary of these searches is presented in Table 1.

**Table 1.** Studies relating to “interstitial pregnancy” between 2005 and 2010, found in the PubMed, Cochrane Library, Lilacs and Embase databases. These searches were made on July 30, 2010, for the first three databases, and on February 23, 2011, for the last of these databases

Database	Terms used	Results	Type of article
PubMed	“pregnancy, ectopic” in Medical Subject Headings AND “interstitial” in all terms	8	Case report
		2	Prospective study
		8	Review
		1	Retrospective study
		1	Comparative study
Cochrane Library	pregnancy AND ectopic AND interstitial	0	
Embase^*^	pregnancy AND ectopic AND interstitial	2	Case report
		1	Prospective study
		1	Review
Lilacs^†^	“gravidez ectópica” in Medical Subject Headings and “intersticial” in words	1	Review

Embase = Excerpta Medica Database; Lilacs = Literatura Latino-Americana e do Caribe em Ciências da Saúde; ^*^Two case reports,^23^^,^^24^ one prospective study^22^ and one review^7^ already selected in PubMed database; ^†^already selected in PubMed database.^7^

Since interstitial pregnancy is a rare form of ectopic pregnancy, conducting a randomized study would be problematic. Hence, case studies documenting the experiences of different medical teams are important, since they facilitate future reviews of the evidence and add to the medical knowledge base pertinent to this condition. Thus, documentation of more cases will enable analysis across cases and aid in establishing improved medical practices.

## CONCLUSION

In the case presented here, methotrexate was not effective for treating an interstitial pregnancy. Persistent embryonic cardiac activity indicated a second dose, without success. Resection was done successfully using video laparoscopy.
